# Screening and Validation of Reference Genes for Normalization of qRT-PCR in Rice BLB Pathogen *Xanthomonas oryzae* pv. *oryzae* Under Tetramycin Stress

**DOI:** 10.3390/genes16070788

**Published:** 2025-06-30

**Authors:** Feiyan Fang, Xinli Miao, Tong Mou, Zian Wang, Yanhe Guo, Yingfen Yang, Shunyu Gao, Zhenji Wang, Chengdong Xu, Jun Yang

**Affiliations:** 1 College of Resources, Environment and Chemistry, Chuxiong Normal University, Chuxiong 675000, China; 15758595063@163.com (F.F.); 18869057131@163.com (T.M.); 18788276629@163.com (Z.W.); 18753809280@163.com (Y.G.); yangyf87@163.com (Y.Y.); gsy88@cxtc.edu.cn (S.G.); wangzj@cxtc.edu.cn (Z.W.); chtown@cxtc.edu.cn (C.X.); 2 School of Mathematics and Statistics, Chuxiong Normal University, Chuxiong 675000, China; miaoxinli@cxtc.edu.cn

**Keywords:** *Xanthomonas oryzae* pv. *oryzae*, tetramycin, real-time fluorescence quantitative PCR, reference gene, quantitative analysis

## Abstract

Background: *Xanthomonas oryzae* pv. *oryzae* (*Xoo*) causes rice leaf blight (BLB) and poses a major threat to global rice production. In rice production, tetramycin agents provide good control of rice leaf blight, while the standardization of the reference genes of *Xoo* under tetramycin stress has not been reported. The aim of this study was to identify the most stable reference genes for quantitative PCR analysis of *Xoo* under tetramycin stress. Methods: Six candidate reference genes, *gyrB* (RNA polymerase *β* gene), *GADPH* (glyceraldehyde-3-phosphate dehydrogenase gene), *recA* (recombinase A gene), *gyrA* (citrate synthase encoding gene), *dnaK* (molecular chaperone protein gene), and *16S rRNA* (*16S* ribosomal RNA gene) were selected and their expression stability was assessed under tetramycin stress conditions using real-time quantitative PCR (qRT-PCR). GeNorm, NormFinder, BestKeeper and RefFinder were used to assess the expression stability, the relative expression values of the eight genes involved QS (Quorum sensing) pathway under tetramycin stress were used to validate by the *rpf* (regulation of pathogenic factors) gene family. Results: *16S rRNA* expression was most stable under tetracycline stress, whereas *GADPH* was the least. The *rpf* gene family showed a highly stable expression level, confirming the reliability of *16S r RNA* as a reference gene in the study of *Xoo* under tetramycin stress. Conclusions: *16S rRNA* was identified as the best reference gene for *Xoo* gene expression analysis under tetramycin stress. It provides a reliable support for the molecular research on the control strategy of rice BLB.

## 1. Introduction

Rice serves as the main food source for over half of the world’s population [1]. However, BLB disease caused by *Xoo*, is a major threat to rice production [2]. The disease causes grey-brown blight and disrupts photosynthesis by colonizing the vascular system of leaves. As this disease spreads, it affects rice yields, and reductions can range from 10% to 30%. In severe cases, losses may reach up to 50% or even result in complete grain failure in heavily affected areas [3,4]. With climate change and the mutation of pathogens, *Xoo* has expanded from traditional rice-growing regions to highland areas, posing a major risk to sustainable rice production in China [5]. 

The control of BLB in rice planting is mainly based on breeding resistant varieties and antimicrobial agent control [6]. Planting disease-resistant varieties is the most cost-effective method for controlling this disease [7]. However, the rapid evolution of pathogenic bacteria can lead to the gradual loss of the original resistance in these resistant varieties. Additionally, the process of breeding new resistant varieties is lengthy [8]. Antimicrobial agents are frequently used in production due to their low cost and quick effectiveness. Controlling bacterial diseases in crops mainly involves the use of inorganic Cu^2+^ compounds, organosulfur compounds, amides, heterocyclic compounds like thiazoles, and agro-antibiotics [9,10,11,12,13,14,15]. In our previous study, tetramycin has broad-spectrum antimicrobial properties and is used in controlling various crop diseases.

Tetramycin is produced by the metabolic fermentation of *Streptomyces hygroscopicus* [16]. It is a 26-member tetraene antibiotic and functions as a broad-spectrum agent antibiotic [17]. It includes four types of antibiotic compounds: Components A1 and A2 are macrolide tetracyclic antibiotics, component B is a peptide antibiotic, and component C is a nitrogen-containing heterocyclic aromatic antibiotic [18]. Studies have found that a 0.3% tetramycin aqueous solution (AS) exhibited excellent antibacterial activity against *Pseudomonas syringae* pv. *actinidiae*. Additionally, the 0.3% tetramycin AS showed effective control in the field against canker, soft rot, blossom blight, and brown spot disease in kiwifruit [19]. It is worth noting that tetramycin has demonstrated excellent effectiveness in controlling the bacterial leaf blight of plateau japonica rice varieties “Chugeng”, both in greenhouse and field conditions [20]. The research indicates that bactericides like tetramycin were effective preventive measures in field trials. However, repeatedly applying the same treatment may accelerate the development of resistant strains and contribute to the formation of resistance [21]. This situation emphasizes the necessity for more comprehensive research into the mechanisms of agents against bacterial pathogens. 

To control crop diseases with antimicrobial agents, it is key to identify pathogen targets by analyzing gene expression changes in pathogens exposed to these agents [22]. qRT-PCR is a precise and sensitive gene expression analysis method. It is more advanced than traditional PCR, offering high specificity, sensitivity, and real-time detection, making it a top choice for plant science research [23]. Reference genes, which have stable expression across different conditions, are essential for accurate target gene expression measurement and experimental variable correction [24,25].

The accuracy of qRT-PCR technology relies on the stability of the reference gene. For effective qRT-PCR analysis, reference genes must exhibit consistent expression across different stress conditions. Maintaining consistency is crucial for correcting variations in RNA loading and reverse transcription efficiency, thereby ensuring the accuracy of the results [26]. In the study of gene expression, selecting appropriate reference genes is essential for accurate gene expression analysis. The selection of reference genes is not routine, it must be determined based on the specific experimental conditions and species characteristics [27]. Commonly used reference genes in *Xoo* research includes *gyrB*, *GAPDH*, *recA*, *gyrA*, *dnaK*, *16S rRNA*, and other housekeeping genes [28,29,30,31,32]. Selecting appropriate reference genes is essential for accurately analyzing gene expression in *Xoo*. The *gyrB* gene showed high expression stability during the early stage of bacterial infection, but showed significant variation under oxidative stress [33]. Although the expression of the *GAPDH* gene is relatively stable in the basic metabolism pathway of bacteria, its expression level may change under different host transformations or nutritional conditions [34]. The expression of *dnaK* gene is relatively stable in response to the stress of bacteria, but its expression stability can differ among various strains or host environments [35]. The *16S rRNA* gene, often used as a reference gene in prokaryote studies, is relatively stable across different growth stages and experimental conditions. Nevertheless, expression level can still exhibit variability between different strains [36]. The existing reference system struggles to meet the precise needs of studying the interaction between antimicrobial agents and pathogens due to instability. Therefore, it is crucial to identify stable reference genes expressed by *Xoo* under tetramycin stress. This identification will help in understanding the effects of antibacterial agents and pathogens.

To analyze the expression stability of candidate reference genes, the average Ct value for each sample can be calculated using the Delta CT method. By combining the analysis results of three commonly used software geNorm, BestKeeper, and NormFinder, the expression stability of candidate reference genes can be comprehensively evaluated by the online software RefFinder [37,38,39]. However, using a single reference gene may not be sufficient to ensure accuracy, and incorporating two or more reference genes can enhance the reliability of the results. In the experiment with very high requirements for quantitative accuracy of gene expression, selecting two stability genes as reference genes can improve the accuracy of the experimental results and ensure the reliability of the relative expression results of the target genes [40]. However, there have been no reports on reference genes for stable expression analysis in *Xoo* under antimicrobial agent stress.

The QS mechanism is crucial for the adaptability and pathogenicity of pathogens [41]. The *rpf* gene family influences the group behavior and pathogenicity of pathogenic bacteria by regulating the diffusible signal factor (DSF) [42]. Consequently, this study systematically assessed the expression stability of six candidate reference genes (*gyrB*, *GAPDH*, *recA*, *gyrA*, *dnaK*, and *16S rRNA*) for *Xoo* strains under tetramycin stress. Various algorithms, including geNorm, BestKeeper, and NormFinder, were employed for joint analysis, while RefFinder was utilized for a comprehensive evaluation to identify the most stable reference genes under bactericide stress in *Xoo*. On this basis, the expression of the *rpf* gene family was verified through quantitative reverse transcription polymerase chain reaction (qRT-PCR), and the response mechanism of *Xoo* under pesticide stress was elucidated, thereby providing a theoretical foundation for the development of innovative prevention and control strategies.

## 2. Materials and Methods

### 2.1. Test Strains

Test strains: The LF21-38 strain of *Xoo* was isolated from the japonica rice cultivar *Chugeng 49* in Jinshan Town, Chuxiong Prefecture, China, (102.063 °E, 25.168 °N). This strain has been identified as the predominant genotype strain in Chuxiong prefecture, located in the central part of Yunnan Province. The complete genome sequencing of the *Xoo* LF21-38 strains has been finished (data unpublished), and the strain is preserved at −80 °C, in the Biochemical and Molecular Laboratory of the College of Resources, Environment, and Chemistry at Chuxiong Normal University. The drug-sensitive test of the *Xoo* LF21-38 strain was tested for the zone of inhibition as previously described [43]. In the previous experiment, the minimum inhibitory concentration (MIC) of tetramycin was 3 × 10^−6^ mg/L. The filter paper diffusion method was slightly improved. Firstly, a bacterial suspension of 3 × 10^8^ cfu/mL was prepared, and the absorbance was measured to be about 0.5 at λ = 600. The prepared bacterial suspension was uniformly coated on the surface of the Nutrient Agar (NA, Peptone 0.5 %, beef extract 0.3 %, yeast extract powder 0.1 %, glucose 1 %, agar 1.7 %, pH 7.0) medium. After soaking the filter paper with a diameter of 0.5 cm for about 30 min in each concentration gradient, the filter paper containing the drug was taken with a bamboo stick and transferred to the surface of the NA medium. Sterile water was used as a blank control. Each group of tests was repeated three times and cultured in a constant temperature incubator at 28 °C for 2-3 days. The diameter of the inhibition zone was observed and recorded. Inhibition rate = (diameter of inhibition zone-diameter of filter paper)/diameter of filter paper. [44].

### 2.2. Selection of Reference Genes and the Design of Primers 

Candidate reference genes were screened from the *Xoo* genome database. Based on the requirements of qRT-PCR reaction for primer specificity, the qRT-PCR upstream and downstream primers of candidate reference genes were designed using Primer Premier 5.0. The primers were synthesized by Beijing Qingke Biotechnology Co., Ltd. Some genes used the published sequence as a reference, the primer sequence is listed in Table S1. After PCR-specific amplification, six relatively stable candidate reference genes were initially selected. In qRT-PCR, the specificity of primers can be judged by the melting curve. If the melting curve shows a single peak shape, it usually means that the primers have good specificity [45]. Amplification efficiency (E) and the Correlation correlation R^2^ of primers can be determined by generating standard curves. The mean CT value (y) for each dilution (x) in the cDNA dilution series was calculated, and the slope of the regression line was determined using the log10 values. Amplification efficiency (E) was calculated using the formula E = (10^[−1/slope]−1) ×100%. Typically, an amplification efficiency of 90-110% is considered optimal [46].

### 2.3. Extraction of RNA and Synthesis of cDNA

Total RNA extraction and cDNA synthesis of pathogens and pathogens treated with fungicides. The total RNA of *Xoo* LF21-38 treated with the MIC of 3 × 10^−6^ mg/L (added to the pathogen in the logarithmic growth phase) and *Xoo* LF21-38 treated with sterile water were extracted by RNA prep pure culture cell/bacterial total RNA extraction kit, respectively. RNA integrity was assessed through 1% agarose gel electrophoresis, while RNA concentration and purity were determined using an ultra-microUV-visible spectrophotometer. Samples meeting the criteria of an A_260_/A_280_ ratio between 1.8 and 2.2 were considered suitable for further experiments. Following the protocol outlined in the BeyoRT™ III First-Strand cDNA Synthesis Kit, 1 μg of total RNA was utilized for reverse transcription to generate cDNA, which was subsequently stored in a -20 ℃ freezer for future applications.

### 2.4. qRT-PCR Analysis

qRT-PCR reaction conditions. According to the instructions of the Real Master Mix SYBR Green kit, qRT-PCR was performed using a real-time quantitative PCR instrument. 20 μL reaction system: 2 × SuperReal PreMix Plus 10 μL; 1 μL forward and reverse primers (10 μmol/L); cDNA 0.5 μL; 50 × ROX Reference Dye 2 μL; each set of three biological replicates. Each sample was subjected to 3 biological replicates. The 2^−ΔΔCt^ method was used to determine the relative fold change in reference gene expression post-amplification. Primer specificity was confirmed through melting curve analysis.

### 2.5. Reference Gene Screening

The expression stability of candidate reference genes was Evaluated, Microsoft Excel 2003 was used to count and process the obtained CT values. The stability of the six candidate reference genes was evaluated using the Delta CT method in geNorm (Biogazelle and Microsoft Excel geNorm), NormFinder (developed by the Department of Molecular Medicine, Aarhus University Hospital, Denmark (MOMA) and NormFinder.xla, 0953 version), BestKeeper version 1 (developed by Michael W. Pfaffl and his team and BestKeeper), and Microsoft Excel 2019. In the geNorm algorithm, the stability of each gene is evaluated by calculating the expression stability value (M) of each gene. The lower the M value, the more stable the expression of the candidate reference gene [36]. NormFinder uses a model-based analysis to assess the expression changes of the candidate endogenous genes and assigns a stability value to each candidate gene. The lower the stability value, the more stable the expression of the candidate reference gene [39]. BestKeeper calculated the standard deviation (SD) of candidate reference genes by analyzing the original data (CT value). The smaller the standard deviation (SD), the more stable the expression of the candidate reference gene The Delta CT method calculates the mean standard deviation (SD) by comparing the CT values of candidate genes in pairs. Among them, genes with smaller SD values are considered to be more stable in expression [47]. Finally, the RefFinder website (http://blooge.cn/RefFinder/, accessed on 24 May 2025) was used for a comprehensive evaluation. RefFinder is a network-based, comprehensive analysis tool specifically for evaluating and screening candidate reference genes. It integrates four commonly used computational programs (geNorm, NormFinder, BestKeeper, and Delta CT) to comprehensively rank candidate reference genes. In the process of analysis, RefFinder assigns corresponding weights to each candidate gene according to the ranking results of each program, and calculates the geometric mean of these weights to obtain the overall ranking of genes [48].

### 2.6. Validation of Reference Gene Stability

In order to verify the stability of the selected reference gene, the wild-type strain *Xoo* LF21-38 was cultured under the tetramycin (with the minimum inhibitory concentration) and sterile water, respectively. Each experimental group was repeated three times and cultured in a constant temperature incubator at 28 °C to determine the bacteriostatic effect of tetramycin. Furthermore, the most stable *16S rRNA* and the most unstable *GADPH* were used as reference genes for real-time fluorescence quantitative PCR. The *Xoo rpf* gene primers are listed in Table S2, and the relative expression of the quorum-sensing-related *rpf* gene cluster *(rpfA-H*) related to quorum sensing was quantified to confirm the stability of the reference gene. This provides an experimental basis for the subsequent comprehensive analysis of the strain.

### 2.7. Statistical Analyses

Gene expression levels were determined through the 2^−ΔΔCt^ method and statistically analyzed using IBM SPSS Statistics 22.0. Significance analysis was assessed using Duncan’s test, and GraphPad Prism 9.0 was utilized for data visualization.

## 3. Results

### 3.1. Validation of Primer Specificity for Candidate Reference Genes

The A_260_nm/A_280_nm ratio of total RNA in both samples fell within the range of 1.9 to 2.0 The total RNA mass is shown in Figure S1 and Table S3, where the two ribosomal components, *23S rRNA* and *16S rRNA*, are clearly distinguishable on the agarose gel. The intensity of the *23S rRNA* band is approximately double that of the *16S rRNA* band, and there is no apparent degradation, suggesting that the sample possesses good integrity and is suitable for subsequent cDNA synthesis.

PCR confirmed the specificity of six candidate reference genes. The amplified bands of the candidate reference genes were within the expected size range of 100~300 bp, demonstrating consistency with the pre-design. The bands appeared clear, singular, and devoid of primer dimers (Figure S2). Subsequent qRT-PCR analysis revealed single-peak dissolution curves for the candidate reference genes, indicating the absence of non-specific amplification. Amplification efficiencies fell within the range of 98.7% to 112.2% (Figure S3) meeting the acceptable threshold of 90-110% (Table S4). These findings suggest that the chosen primers exhibit high specificity and are well-suited for qRT-PCR analysis.

### 3.2. CT Value Analysis

**Figure 1:**
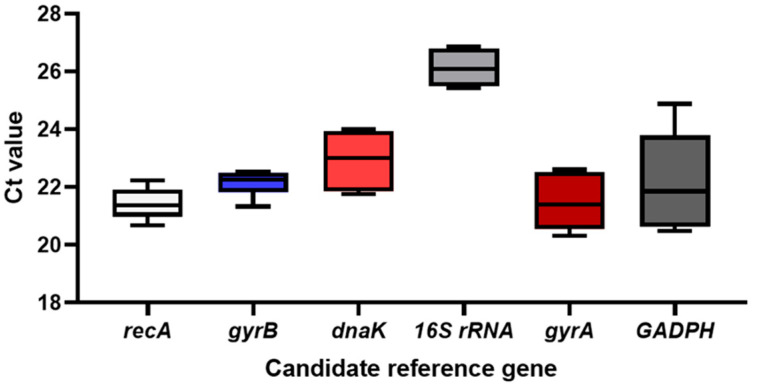
(CT value) expression levels of six reference genes in *Xoo.* Note: The box represents the centralized range of Ct values, the black square in the center of the box represents the median, and the upper and lower ends of the box represent the maximum and minimum values, respectively.

The CT value serves as an indicator of the expression level of a reference gene. Generally, a lower CT value corresponds to a higher initial gene copy number, indicating a higher expression level. Conversely, a higher CT value indicates a lower initial gene copy number and a lower expression level. Analysis of the box plot displaying the CT values of six candidate reference genes (Figure 1, Table S5) reveals that *recA*, *gyrB*, *gyrA*, and *GAPDH* exhibit relatively small CT values, while *dnaK* and *16S rRNA* display larger values. Regarding expression levels, *recA*, *16S rRNA*, *dnaK*, and *gyrA* demonstrate a narrow fluctuation range, whereas *gyrB* and *GAPDH* exhibit a broader range. In terms of distribution, *recA*, *16S rRNA*, *dnaK*, and *gyrA* show a more concentrated distribution, whereas *gyrB* and *GAPDH* are more dispersed. Consequently, it can be tentatively concluded that *recA*, *16S rRNA*, *dnaK*, and *gyrA* exhibit greater stability in expression levels compared to other genes and are suitable as candidate reference genes.

### 3.3. Delta Ct Values Analysis

**Figure 2:**
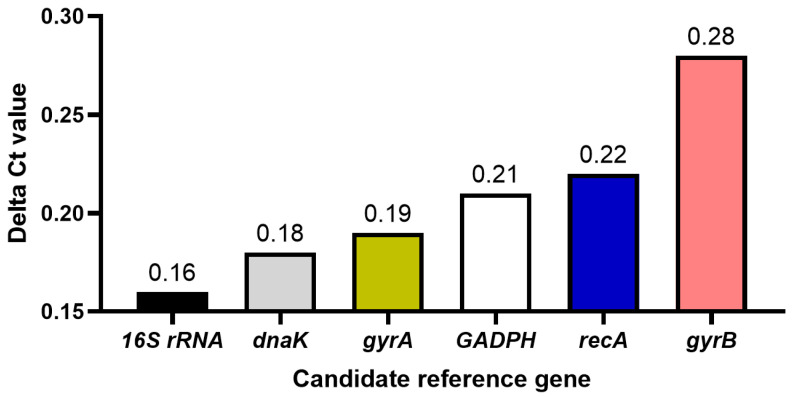
Delta CT results of the six candidate reference genes.

Candidate reference gene expression stability analysis. Analysis of Delta Ct values: The relative expression of the six candidate reference genes under control and experimental conditions was evaluated. The average Ct value of each amplicon in each sample was calculated using the original Ct value. The results showed that (Figure 2), among the six candidate reference genes, the expression of the 16S rRNA gene was the most stable, followed by the dnaK gene, and the most unstable expression was the gyrB gene.

### 3.4. GeNorm Analysis

**Figure 3:**
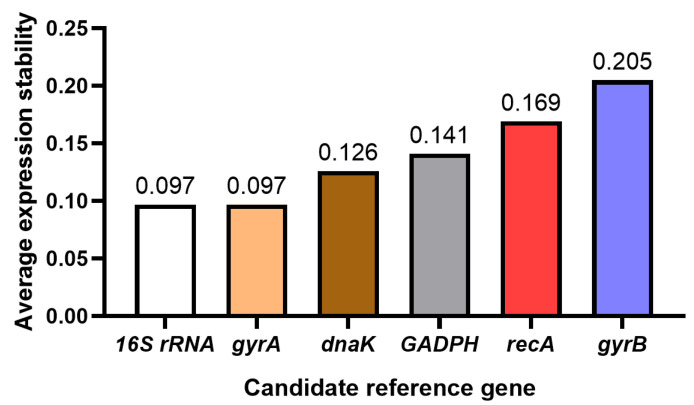
GeNorm analysis of the expression stability of candidate reference genes.

GeNorm was used to analyze the stability of six reference genes under tetramycin stress. The better the stability of the gene, the lower the average expression stability index (*M* value). The calculation method of the *M* value is to compare the expression levels of a reference gene with other reference genes, and then logarithmically convert these ratios and calculate their average standard deviations. If the *M* value exceeds 1.5, this indicates that the expression of the gene is not stable enough, so it is not suitable as a reference gene. In short, genes with an *M* value of less than 1.5 are considered to be stable and suitable as reference genes. The expression stability *M* values of the genes were calculated, and the expression stability of the genes was sorted according to the *M* value. The results showed that the more stable reference genes were *16S rRNA* and *gyrA*. The *M* values of four candidate reference genes were lower than 0.25, which could be listed as candidate reference genes. that is, *16S rRNA*, *gyrA*, *dnak*, and *GADPH* (Figure 3).

### 3.5. Normfinder Analysis

**Figure 4:**
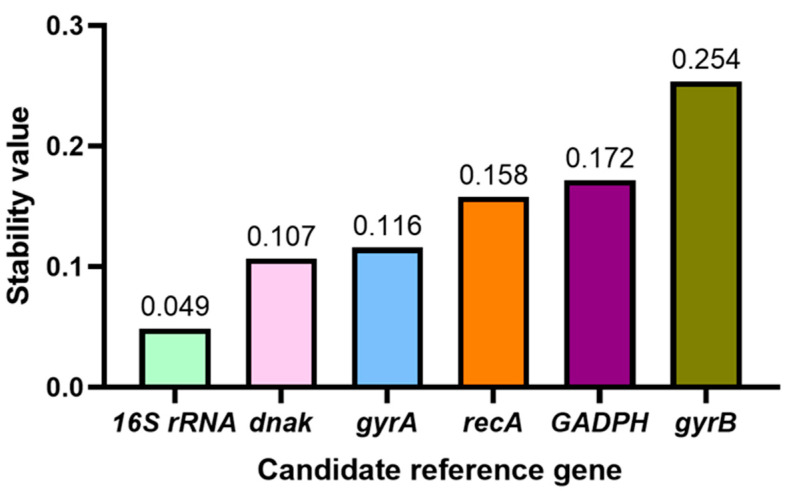
Normfinder analysis of the expression stability of candidate reference genes.

NormFinder is a method for assessing the stability of reference genes, which calculates a Stability Value (SV) by analyzing the differences in expression of a reference gene between and within different sample groups. The smaller the stability value, the more stable the expression of the reference gene. By comparing the SV values of each reference gene, they can be sorted to find the gene with the smallest SV value, which is the optimal reference gene. Normfinder was used to analyze the stability of six reference genes under tetramycin stress (Figure 4). The most stable gene expression was *16S rRNA*, followed by *dnak*, and the worst stability was the *gyrB* gene.

### 3.6. BestKeeper Analysis

BestKeeper is a tool to evaluate the stability of reference genes by analyzing the threshold cycle (Ct value) of each reference gene. Using the Ct values, the standard deviation (SD) of the expression levels of these genes can be calculated. Standard deviation is an indicator of the degree of dispersion of the data distribution. The level of SD value directly reflects the stability of gene expression; the lower the SD value, the more consistent the gene expression, and the more suitable it is as a reference gene. The BestKeeper software was used to screen the reference genes under tetramycin stress. The stability of gene expression shown in Table 1 was *gyrB > recA > 16S rRNA > gyrA > dnak > GADPH* from high to low. In addition, in order to evaluate the relationship between all candidate reference genes, BestKeeper can also perform pairwise correlation analysis. The Pearson correlation coefficient r is shown in Table 1. *GADPH* has the highest correlation with other genes, followed by *16S rRNA* and *dnak*. These three genes also reached a very significant level (*P* < 0.01), and the correlation of *gyrB* was the lowest. 

### 3.7. RefFinder Analysis

The above different software analysis results are not the same, which may be the result of algorithm differences. The RefFinder website is a comprehensive online tool that uses a variety of software algorithms, including NormFinder and BestKeeper, and the Delta Ct method, to evaluate and rank the stability of candidate reference genes. This website combines the results of these different methods and uses the geometric mean method to provide a comprehensive stability ranking. The RefFinder website provides users with a convenient and quick way to determine which reference genes are most stable by integrating a variety of evaluation methods RefFinder. RefFinder is used to comprehensively analyze the results of the above three software and the Delta Ct. The ranking of geNorm, Normfinder, and BestKeeper on the website is consistent with the stability ranking results obtained by using the above separate programs. From the comprehensive ranking, among the six candidate reference genes, *16S rRNA* had the highest expression stability, followed by *gyrA*, and *GADPH* had the lowest stability (Table 2). Therefore, *16S rRNA* was suitable as a reference gene for gene expression of *Xoo* under the minimum inhibitory concentration stress of tetracycline.

### 3.8. Validation of Reference Gene Stability

**Figure 5:**
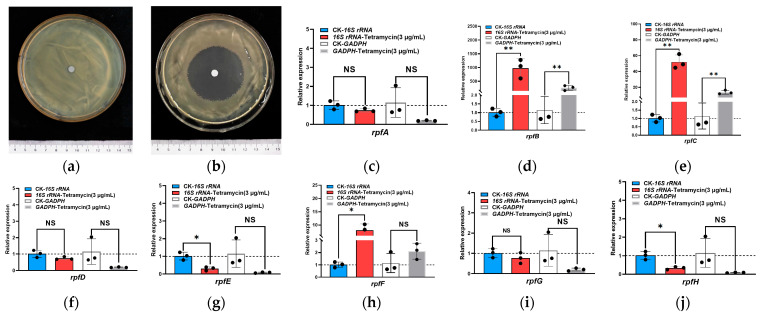
Analysis of the quorum-sensing-related gene expression in *Xoo* under tetramycin stress. Note: (**a**) The drug sensitive test of *Xoo* strains LF21-38 under the tetramycin with minimum inhibitory concentration; (**b**) The drug sensitive test of *Xoo* strains LF21-38 under sterile water as control; (**c**) relative quantitative results of *rpfA*; (**d**) relative quantitative results of *rpfB*; (**e**) relative quantitative results of *rpfC*; (**f**) relative quantitative results of *rpfD*; (**g**) relative quantitative results of *rpfE*; (**h**) relative quantitative results of *rpfF*; (**i**) relative quantitative results of *rpfG*; (**j**) relative quantitative results of *rpfH*. (“*” Significant, *p* < 0.05, “**” Very significant, *p* < 0.01, “ns” unsignificant).

The drug sensitivity test results indicated that, in comparison to the sterile water control group, the LF21-38 strain exhibited a significantly larger inhibition zone diameter under the minimum inhibitory concentration of tetramycin (Figure 5a,b), with inhibition rates ranging from 88% to 89%, the diameter is between 4.4–4.6 cm. (Table S6). Notably, the inhibition rate reached 89% at the minimum inhibitory concentration of tetramycin, suggesting enhanced sensitivity of the strain to this antibiotic. Based on this, the expression of the quorum sensing related *rpf* gene family in response to tetramycin was verified by qRT-PCR using the most stable internal reference gene, *16S rRNA* (Table S7). The results indicated that the expression of QS related genes in *Xoo* was significantly up-regulated following tetracycline treatment. The relative expression quantities were calculated in this experiment using the *16S rRNA* gene as the reference standard, the expression levels of *Xoo* QS related genes *rpfA*, *rpfD*, and *rpfG* genes were not significantly affected by tetramycin stress. In contrast, the expression levels of *rpfB*, *rpfC*, and *rpfF* genes were significantly up-regulated, with an increase of 976.32-fold, 52.00-fold, and 7.98-fold, respectively. Additionally, the expression of the *rpfE* and *rpfH* genes was significantly reduced (Figure 5c–j). When using the *GADPH* gene as the reference gene, the expression of the *GAPDH* gene was found to be unstable, showing significant fluctuations over a wide range, when comparing the expression levels of each gene with the *16S rRNA* gene as the reference group, significant differences were observed. For instance, *rpfF* is a crucial factor in QS and is essential for participation in this process. In contrast, when using the *GAPDH* gene as the reference group, no significant expression was detected. With the *16SrRNA* as the internal reference gene, the fluctuation range was minimal, and the expression level was consistent with previous reports. Consequently, *16S rRNA* gene is identified as the most stable reference gene expressed in the *Xoo* strain under tetramycin stress. This further suggests that tetramycin-induced stress affects the communication of quorum-sensing signaling molecules. Therefore, it can be inferred that quorum sensing may play a role in the *Xoo* strain’s response mechanism to tetramycin.

## 4. Discussion

In gene expression analysis, selecting an appropriate reference gene is a crucial part of the experimental standardization process. The stability of this reference gene’s expression directly affects the reliability of the corrections made to target gene expression [47]. One of the key factors ensuring the reliability of experimental data is the stability of the reference gene [48]. However, even within the same species, the expression stability of a reference gene can vary under different physiological conditions, and it may not always be consistent. In addition, reference genes can exhibit different stability performances across different species. Therefore, it is particularly important in specific studies to identify the most stable reference genes that are best suited for the target species [49].

In the qRT-PCR experiment, standardizing the geometric mean of multiple reference genes is crucial for ensuring data accuracy. This strategy is suitable for various gene and tissue samples and can significantly enhance the detection accuracy of small expression differences, providing a more reliable basis for biological research [50]. Key factors affecting quantitative gene expression analysis include RNA quality, the cDNA reverse transcription process, and PCR amplification conditions [51]. By carefully managing and optimizing various factors, we can enhance the accuracy and reliability of gene expression analysis. In this study, we utilized several statistical methods to thoroughly assess the expression stability of potential reference genes. Our results indicated that different analytical methods could yield varying stability rankings, likely due to the differences in their underlying algorithms. Using a single reference gene for normalization can lead to significant errors in some samples. In contrast, utilizing the geometric mean of multiple reference genes can substantially enhance the accuracy of normalization. The geNorm program evaluates various reference genes in experiments and identifies at least two optimal combinations for data correction, thus enhancing the accuracy of relative quantitative results by analyzing the variance between groups and within groups, NormFinder identities genes that are stably expressed under different experimental conditions, thereby improving the accuracy and reliability of qRT-PCR data [38]. BestKeeper not only screens for stable reference genes, but also evaluates the integrity of the sample, providing a solid foundation for selecting suitable reference genes for qRT-PCR experiments, thus enhancing the reliability of gene expression analysis [25]. However, RefFinder is a comprehensive assessment tool that provides more consistent and reliable results for ranking reference genes by integrating multiple evaluation methods. This method allows for a more thorough assessment of the stability of the reference gene and reduces the potential deviation that may occur when relying on a single method [52]. The RefFinder software incorporates geNorm, NormFinder, BestKeeper, and Delta-CT methods, and its ranking on the website aligned with the stability ranking obtained by using each method independently [53]. From the combined ranking, the two candidate reference genes with the highest expression stability were *16S rRNA* and *gyrA*, making them suitable choices for reference genes in studies of gene expression related to rice bacterial leaf blight pathogens (*Xoo*) under the stress of minimum inhibitory concentration of the agent tetramycin. 

To investigate the expression of functional genes in bacteria during different growth stages or conditions, *16S rRNA* is mostly used as a reference gene. The *16S rRNA* sequence is highly conserved and specific in most bacteria, making it a common choice as a normalized reference gene [54]. Research evaluated eight candidate reference genes in *Pseudomonas fluorescens* cultured with different exogenous signaling molecules. Analysis using geNorm, NormFinder, BestKeeper, and RefFinder indicated that the *16S rRNA* gene had excessively high expression and low stability, making it unsuitable as a reference gene [55]. The results of this study showed that *16S rRNA* exhibited the most stable characteristics after verification with various algorithms, making it the best reference gene for *Xoo*. It is further explained that the research conclusions of one bacterium cannot be simply applied to the research of another bacterium, and the appropriate reference genes should be determined according to the specific bacteria and research conditions.

In addition, this study highlighted the importance of considering specific experimental conditions when selecting reference genes. *GADPH*, which encodes glyceraldehyde-3-phosphate dehydrogenase, serves as a reference gene for evaluating gene expression by qRT-PCR in various bacterial species. In contrast, a study on *Xanthomonas campestris* pv. *campestris* (*Xcc*) assessed multiple candidate reference genes and found that *GADPH* expression was unstable, making it unsuitable as a reference gene [56]. This is consistent with the results of the *GADPH* screening in this study. Reference genes play a key role in ensuring the stability and accuracy of gene expression analysis. Using inappropriate reference genes may lead to deviations and misleading results. Therefore, it is essential to screen and verify multiple candidate reference genes to ensure that the selected ones demonstrate stable expression levels under specific experimental conditions.

The analysis of key signaling pathways in microbial QS highlights that *rpf* gene family is important for *Xanthomonas* spp. pathogen virulence and environmental adaptation [57]. *Xcc* interacts with host plants, prompting them to produce salicylic acid (SA), which affects the quorum sensing system of *Xcc*, altering the transport and virulence of signaling molecules from the DSF family in a way that depends on the *rpf*B gene [58]. In this study, the most stable *16S rRNA* was used as a reference, and the expression of the *rpfB* gene in *Xoo* was significantly increased under tetramycin stress, emphasizing its important role in regulating gene expression in response to external stress factors. The *rpfF* gene is essential for maintaining the cell membrane barrier; mutations can compromise this barrier and increase bacterial vulnerability to external stress [59]. Among them, *rpfF* is involved in mediating the expression of *rpfB* and is a key enzyme in the synthesis of QS signal molecules. Our study found that under tetramycin stress, the *rpfF* gene is significantly expressed, indicating that bacteria may boost their expression to enhance their membrane barrier and improve resistance to tetramycin. Under different nutritional conditions, the *rpfE* gene can affect pathogenicity by regulating the pathogenic factors of *Xoo* and the efficiency of carbon source utilization, without involving changes to DSF signals [60]. However, in this study, the expression of the *rpfE* gene was down-regulated, which indicated that the drug may have an inhibitory effect on the expression of the *rpfE* gene under the stress of tetramycin.

In summary, the *rpf* gene family plays an important role in regulating the virulence and environmental adaptability of pathogenic bacteria under normal conditions. In this study, we observed that the expression patterns of several genes within the *rpf* gene family changed significantly in response to tetramycin. Specifically, genes such as *rpfB*, *rpfF*, *rpfC*, *rpfG*, *rpfH*, and *rpfE* were either up-regulated or down-regulated. This suggests that tetramycin may disrupt the signal transduction and gene regulation networks within bacteria, altering the normal regulatory mechanisms of the *rpf* gene family. As a result, this could affect the pathogenicity and environmental adaptability of these bacteria. It is speculated that the *rpf* gene family may have complex regulatory changes and potential response mechanisms when under to tetramycin stress.

Therefore, this study investigated the relationship between signal regulation expression and the function of QS signal-related genes in the *rpf* gene family regarding drug sensitivity. Studying the interaction mechanisms between drugs and pathogen targets is essential. By establishing a standardized reference system and selecting the most stable reference gene, we can analyze the relative expression of *Xoo* under stress from tetramycin. This approach provides a theoretical basis for developing new prevention and control strategies that focus on inhibiting QS. The results of this study have important significance for the study of *Xoo*, but also provide valuable insights for gene expression research in other plant pathogens. Future studies can investigate how these reference genes perform under various environmental pressures and their potential applications for other plant pathogens.

## 5. Conclusions

In the wide application of RT-qPCR technology, the stability of internal reference genes has become the core factor to ensure the accuracy of quantitative results. It is necessary to select the appropriate reference gene. In this study, reference genes such as *16S rRNA*, which demonstrated the most stable expression in *Xoo* under tetramycin stress are selected to provide reliable reference of reference genes for the subsequent exploration of *Xanthomonas*, thus obtaining more reliable relative quantitative results in the study of QS gene expression.

## Figures and Tables

**Table 1:** The stability of six candidate reference genes analyzed by Bestkeeper.

Bestkeeper	Means Ct ± Standard Deviation	Correlation Coefficient r	*p*-Value	*Significance*
*gyrB*	22.31 ± 0.151	0.693	0.127	**ns**
*recA*	21.52 ± 0.185	0.876	0.022	**ns**
*16S rRNA*	26.06 ± 0.273	0.986	0.001	**
*gyrA*	21.42 ± 0.298	0.941	0.005	*
*dnak*	23.00 ± 0.330	0.971	0.001	**
*GADPH*	22.58 ± 0.330	0.996	0.001	**

Note: Statistically significant differences are indicated (“*” Significant, *p* < 0.05, “**” Very significant, *p* < 0.01, un significant, “ns”).

**Table 2:** Ranking of expression stability of candidate reference genes.

Order of Precedence	Delta CT	Genorm	Normfinder	Bestkeeper	Overall Ranking
1	*16S rRNA*	*16S rRNA*	*16S rRNA*	*gyrB*	*16S rRNA*
2	*dnak*	*gyrA*	*dnak*	*recA*	*gyrA*
3	*gyrA*	*dnak*	*gyrA*	*16S rRNA*	*dnak*
4	*GADPH*	*GADPH*	*recA*	*gyrA*	*recA*
5	*recA*	*recA*	*GADPH*	*dnak*	*gyrB*
6	*gyrB*	*gyrB*	*gyrB*	*GADPH*	*GADPH*

## Data Availability

The raw data supporting the conclusions of this article will be made available by the authors on request.

## Supplementary Materials

The following supporting information can be downloaded at: https://www.mdpi.com/article/10.3390/genes16070788/s1, Table S1: Primer sequences of candidate reference genes. Table S2: Primer sequences of rpf gene family. Table S3: RNA concentration and quality determination data of LF31-38 strain under sterile water control and tetramycin stress. Table S4: Gradient amplification efficiency of six reference genes Table S5: CT values of six candidate reference genes in the experimental group and the control group. Table S6: Antibacterial rate data determined by filter paper diffusion method. Table S7: Verification of CT value of *rpf* gene expression under reference gene; Figure S1: Agarose gel electrophoresis of total RNA of *Xoo* strain (*Xoo-*CK) and Xoo strain after Ttm treatment (*Xoo*-Ttm). Figure S2: Electrophoresis of PCR products of six candidate reference genes. Figure S3: qRT-PCR analysis curves of the six candidate reference genes.

## Abbreviations

The following abbreviations are used in this manuscript:

*Xoo*	*Xanthomonas oryzae* pv. *oryzae*
Ct	Cycle threshold
Ttm	tetramycin
qRT-RCR	Real-time fluorescent quantitative PCR
QS	Quorum sensing
*Rpf*	Regulation of pathogenicity factors
*gyrB*	RNA polymerase *β* gene
*GAPDH*	glyceraldehyde-3-phosphate dehydrogenase gene
*recA*	recombinase A gene
*gyrA*	citrate synthase encoding gene
*dnaK*	molecular chaperone protein gene
*16S rRNA*	*16S* ribosomal RNA gene
SV	Stability value
SD	Standard deviation
DSF	diffusible signal factor
